# Interdisciplinary investigations into the biophysics of the origins of life

**DOI:** 10.2142/biophysico.bppb-v22.0021

**Published:** 2025-09-12

**Authors:** Tony Z. Jia, Yutetsu Kuruma

**Affiliations:** 1 Office of Research and Academia-Government-Community Collaboration, Hiroshima University, Higashi-Hiroshima, Hiroshima 739-8511, Japan; 2 Blue Marble Space Institute of Science, Seattle, WA, 98104, USA; 3 Institute for Extra-Cutting-Edge Science and Technology Avant-Garde Research (X-star), Japan Agency for Marine-Earth Science and Technology (JAMSTEC), Yokosuka, Kanagawa 237-0061, Japan

The study of the origins of life requires collaboration from researchers of a variety fields, not limited just to biology, chemistry, geology, or planetary science, but also physics, complexity science, and engineering, just to name a few. At the intersection of some of these international collaborations is the field of biophysics, which itself is interdisciplinary in nature. Given the number of different experimental, analytical, and theoretical techniques used by biophysicists, the biophysics field stands in a position where it can contribute to understanding of a significant number of unanswered questions at the origins of life. In Japan, a significant number of biophysicists, especially young researchers, have been contributing to the origins of life field for a number of years [1], partially evidenced by a recent run of annual origins of life symposia on all topics related to the origins of life at the annual Biophysical Society of Japan (BSJ) meeting over the last several years beginning in 2021 [2] (and including at the 2024 joint BSJ/IUPAB (International Union for Pure and Applied Biophysics) meeting, where Ryo Mizuuchi (Waseda University) co-organized a symposium [3]). Building upon these previous symposia, the 2025 annual BSJ meeting held in Nara also includes an Origins of Life Symposium on September 26^th^, 2025 organized by BSJ members Tony Z. Jia (Hiroshima University) and Yutetsu Kuruma (Japan Agency for Marine-Earth Science and Technology (JAMSTEC)), who are experts in prebiotic chemistry/protocells [4,5] and synthetic biology/artificial cells [6,7], respectively.

The theme of the symposium is “Interdisciplinary investigations into the biophysics of the origins of life”, and sought to feature young, promising researchers combining biophysics and at least one other discipline (and in some cases, multiple) to investigate some aspect of the origins of life. Five invited speakers across a variety of disciplines are featured, while one speaker was selected from submitted poster abstracts (Fig. 1). The organizers would like to thank all submitted poster abstracts which could not be selected for oral presentations due to time limitations, and look forward to visiting the posters at the conference.

The first speaker, Paola Laurino of Okinawa Institute of Science and Technology and Osaka University, is an organic chemist and an expert in various aspects of biochemistry. Her recent work on various aspects of membraneless droplets shed new light into the plausibility of membraneless protocells at the origins of life [8,9], and with this scientific base, Paola speaks about “Enzymatic Condensates as self-regulating systems for metabolic efficiency” at the symposium.

The next speaker, Mayumi Seto of Nara Women’s University, has a diverse background in ecology, evolution, and bioenergetics, and is focused on incorporating and discovering thermodynamic rules to various processes at the origins of life, such as microbial metabolism [10,11]. She uses some of these new insights to speak about “Thermodynamic constraints and the choice of metabolic pathways in life” at the symposium.

One of the symposium co-organizers, Yutetsu Kuruma of JAMSTEC, also gives an invited talk titled “The Potential of Artificial Cells Functioning under In Situ Deep-Sea Conditions”, which is based off of a recent study where they subjected vesicles to a deep-sea floor dive in a submarine and allowed them to perform various biological processes, such as protein expression [12].

The next speaker is Jumpei Yamaguchi of the RIKEN Center for Biosystems Dynamics Research, and gives a contributed presentation. His expertise lies in complexity science, and has studied various aspects of evolution and universal biology [13,14]. His talk, titled “Thermodynamic constraint on the coupling of currency metabolites”, uniquely incorporates various aspects of thermodynamics and economics to understand primitive metabolism.

The penultimate speaker, Masaru Nobu of JAMSTEC, is an expert in geobiology and bioinformatics, with a large wealth of knowledge on various Asgard archaea, which may hold the key to the form of the last universal common ancestor (LUCA), as well as the origins of bacterial photosynthesis [15,16]. He contributes a talk titled “Electrons and the origin of three divisions of life”, which focuses on how life could have potentially evolved from LUCA.

Kosuke Fujishima of the Earth-Life Science Institute (ELSI), Institute of Science Tokyo, is the final speaker of the symposium, and is an expert of a wide range of topics including astrobiology, evolution, biochemistry, and synthetic biology [17,18]. Incorporating aspects of prebiotic chemistry, geochemistry, and chemical biology, he gives a talk titled “Supercritical CO_2_ promotes prebiotic nucleotide synthesis via dehydration condensation”, which features a novel undersea system that could have promoted essential prebiotically plausible reactions leading to the origins of life.

We hope to see more and more biophysicists engaged in origins of life research in the future, leading to the continuation of this symposium (organized in collaboration with Korean researchers) at next year’s joint International Conference on Biophysics and Biomedical Sciences (ICBBS)/BSJ meeting in Pusan, South Korea [19] as well as other future meetings. We especially wish to see new symposium organizers emerge, and are looking forward to seeing what great things that the next generation of early career biophysicists in the origins of life field from now!

T.Z.J. is supported by the Mizuho Foundation for the Promotion of Sciences and by Japan Society for the Promotion of Science (JSPS) Grants-in-aid 21K14746 and 25K23395. Y.K. is supported by JSPS Grants-in-Aid 24K02000, 24H02287, and 21H05156, JST CREST (JPMJCR18S6), and the Human Frontier Science Program (RPG0029/2020). The authors would also like to thank the symposium speakers and all poster presenters who submitted their abstracts for this symposium. Please visit their posters and support origins of life research at the BSJ meeting!

Note that due to some sudden unforeseen events, Paola Laurino was not able to speak at the symposium; we hope to hear Paola speak at a future BSJ meeting!

## Figures and Tables

**Figure 1:**
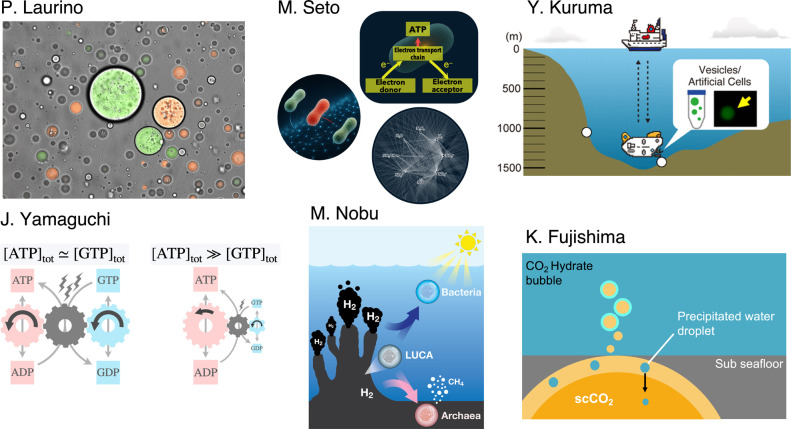
Schematic illustrations representing each author’s presentation.
